# Assessment of risk factors for virological nonsuppression following switch to dolutegravir and lamivudine, or bictegravir, emtricitabine, and tenofovir alafenamide fumarate in a real-world cohort of treatment-experienced adults living with HIV

**DOI:** 10.1371/journal.pone.0314003

**Published:** 2024-11-20

**Authors:** Shu-Yuan Lee, Yi-Chun Lin, Cheng-Pin Chen, Shu-Hsing Cheng, Shu-Ying Chang, Shin-Yen Ku, Chien-Yu Cheng

**Affiliations:** 1 Department of Infectious Diseases, Taoyuan General Hospital, Ministry of Health and Welfare, Taoyuan City, Taiwan; 2 School of Clinical Medicine, National Yang-Ming Chiao Tung University, Taipei City, Taiwan; 3 School of Public Health, Taipei Medical University, Taipei City, Taiwan; 4 Department of Nursing, Taoyuan General Hospital, Ministry of Health and Welfare, Taoyuan City, Taiwan; 5 Institute of Public Health, School of Medicine, National Yang-Ming Chiao Tung University, Taipei City, Taiwan; NIH: National Institutes of Health, UNITED STATES OF AMERICA

## Abstract

Conflicting data exists regarding the baseline determinants of virological nonsuppression outcomes in treatment-experienced people living with human immunodeficiency virus (PWH) switching to antiretroviral treatment (ART) with bictegravir/emtricitabine/tenofovir alafenamide fumarate (BIC/FTC/TAF) or dolutegravir/lamivudine (DTG/3TC) in Asia. This retrospective observational study, conducted at a designated HIV-care hospital from October 2019 to January 2023, aimed to address this gap. We assessed the odds of virological nonsuppression (VNS) at weeks 48 using logistic regression. A total of 988 patients were included, 35 patients (3.5%) with VNS at week 48. Pre-existing primary resistance-associated mutations (RAM) to nucleoside reverse transcriptase inhibitor (NRTI) and non-nucleoside reverse transcriptase inhibitor (NNRTI) were identified in 11.0% (51/465) and 14.4% (67/465), respectively. The identified risk factor was a record of virological failure ≥2 times (AOR 5.32, 95% CI 2.04–13.85), while an HIV viral load <50 copies/mL within the past three months before switch (AOR: 0.27, 95% CI 0.11–0.72) was identified as a protective factor. No cases of acquired drug resistance-associated mutations were detected at week 48. Additionally, DTG/3TC was noninferior to BIC/FTC/TAF in achieving or maintaining HIV RNA levels of <50 copies/mL, within a -10% noninferiority margin in the per-protocol analysis (responder proportion: 98.2% vs. 95.0%, respectively; adjusted treatment difference [95% CI], 3.2% [0.7% to 5.3%]). In conclusion, DTG/3TC and BIC/FTC/TAF demonstrated good effectiveness in a real-world cohort, but frequent virological failure before the switch might impact the benefits of these regimens in the short term of follow-up.

## Introduction

People living with human immunodeficiency virus currently require lifelong antiretroviral therapy, which has significantly improved life expectancy. As a result, HIV infection is now considered one of the communicable chronic diseases. The 2023 guidelines from the US Department of Health and Human Services, the 2023 European AIDS Clinical Society, and the 2022 International Antiviral Society—USA recommend the use of second-generation integrase strand transfer inhibitors (INSTI) as initial antiretroviral regimens. Examples include bictegravir/emtricitabine/tenofovir alafenamide fumarate and dolutegravir/lamivudine for most treatment-naive individuals with HIV and as a switch option for those who are virologically suppressed [1–3]. These recommendations are based on the demonstrated potent, durable antiviral activity and a high barrier to resistance of these medications. Several studies have demonstrated the suppressive efficacy of a two-drug regimen comprising DTG plus 3TC similarly to a tenofovir disoproxil fumarate (TDF) or tenofovir alafenamide (TAF)-based three-drug regimen [4–6].

HIV RNA levels >50 copies/mL at 6 months after starting therapy and confirmed HIV RNA levels >50 copies/mL with previously undetectable HIV RNA levels are defined virological failure [2]. Although INSTI-based ART has been shown to achieve high rates of virological suppression, the incidence of virological nonsuppression commonly ranges from 10% to 40% [7–10]. This includes both low-level viremia (LLV, HIV RNA levels <50–1000 copies/mL) and virological failure (VF, HIV RNA levels >1000 copies/mL). While the risk factors for virological nonsuppression outcomes are not yet fully understood, they may include the transmission route, HIV RNA level, CD4 lymphocyte count, type and duration of ART administered, and poor adherence leading to the emergence of RAMs to antiretroviral agents.

Most studies on second-generation integrase inhibitors predominantly included treatment-naïve or virologically suppressed patients [4–6], and there has been a scarcity of research analyzing the risk factors for virological nonsuppression and the impact of pre-existing resistance-associated mutations among ART-experienced and viremic individuals living with HIV. Therefore, the primary objective of this study is to investigate the risk factors associated with virological nonsuppression outcome at 48-week after switching to DTG/3TC or BIC/FTC/TAF among treatment-experienced patients who were either virologically suppressed or non-suppressed.

## Materials and methods

This retrospective, observational cohort study was conducted at a designated hospital for HIV care in Taiwan. The study was conducted to include the patients aged 18 years or older with confirmed HIV infection who switched their prior ART regimen to DTG/3TC or BIC/FTC/TAF from October 2019 to January 2022, and individuals were followed up until January 2023. There was no limitation on the duration of prior ART, and the regimens consisted of two NRTIs plus an INSTI, NNRTI, or protease inhibitor (PI) to co-formulated DTG/3TC or BIC/FTC/TAF as maintenance treatment. The exclusion criteria were (1) active tuberculosis, (2) allergic history or high degree of sensitivity to any component or auxiliary material of the research drug. (3) positive HBsAg status (4) those who discontinued ART, were lost to follow-up, or passed away from the final analyses. Subsequently, the individuals were switched to DTG/3TC or BIC/FTC/TAF at the discretion of treating physicians, and all included patients were followed for a 48-week period (per protocol analysis).

The aim was to examine baseline factors associated with virological non-suppression outcomes at week 48 after switching to DTG/3TC or BIC/FTC/TAF. Additionally, changes in body weight, total cholesterol, and triglyceride levels over the course of the study will be analyzed in included participants. The study received approval from the research ethics committee or institutional review board (registration number: TYGH112029), and patient consent was waived due to the retrospective nature of the study. The data were accessed for research purposes since August 15, 2023, and all data were analyzed without personal sensitive information.

### Data collection and definition

A standardized case record form was utilized to gather information on demographics, sexual preference, body weight, HIV treatment history, HIV regimens prior to the switch, reasons for switching, HBsAg serology, and results of various laboratory investigations. The tests included plasma HIV RNA level, CD4 lymphocyte count, serum creatinine, liver function, lipid profile, and fasting blood glucose or glycated hemoglobin (HbA1c). These tests were conducted every 3–6 months in adherence to the CDC HIV treatment guidelines in Taiwan, and plasma testing conducted within the past three months before inclusion could serve as reports at the time of switch.

Plasma HIV RNA level was measured using the COBAS AmpliPrep TaqMan HIV-1 test version 2.0 (Roche, Mannheim, Germany), with a lower limit of quantitation of fewer than 20 copies/mL. Virological suppression (VS) was defined as HIV RNA levels <50 copies/mL at week 48, with a 12-week window on either side (snapshot). Low-level viremia was defined as at least twice consecutive plasma HIV RNA level measurements of 50–1000 copies/mL, following VS; viral blip, as an isolated plasma HIV RNA level of 50–1000 copies/mL with previous and subsequent HIV RNA levels <50 copies/mL; and virological failure (VF), as a plasma HIV RNA levels exceeding 1,000 copies/mL. Virological non-suppression included LLV, viral blip, and VF.

Genotypic drug resistance testing was performed routinely since October 2012, but it was not compulsory. All HIV-1 major mutations were identified through population sequencing of HIV-1 RNA and RAMs were predicted using the HIVdb program of the Stanford University HIV Drug Resistance Database, aligning with the drug resistance mutation list of the international AIDS Society-USA Consensus Guidelines [11, 12].

### Statistical analysis

The distributions of patients’ demographics and baseline characteristics were summarized using descriptive statistics. Categorical variables were compared using either the chi-square test or Fisher’s exact test, while continuous variables were assessed using the Mann—Whitney U test.

### Analysis and regression models

A linear regression model was employed to examine the association between changes in weight and lipids, while a logistic regression model was used to assess the risk factors associated with virological non-suppression at week 48. The Hosmer-Lemeshow test and Nagelkerke’s R2 were utilized to assess the proportion of VNS that is explained by the independent variables. All *p* values were two-sided, and statistical significance was set at a *p* value <0.05. Kaplan–Meier survival analysis was employed to assess and compare the time to treatment discontinuation between the two treatment groups. The analyses were conducted using SPSS software version 24.0 (SPSS Inc., Chicago, IL).

## Results

## Patient characteristics

**Fig 1:**
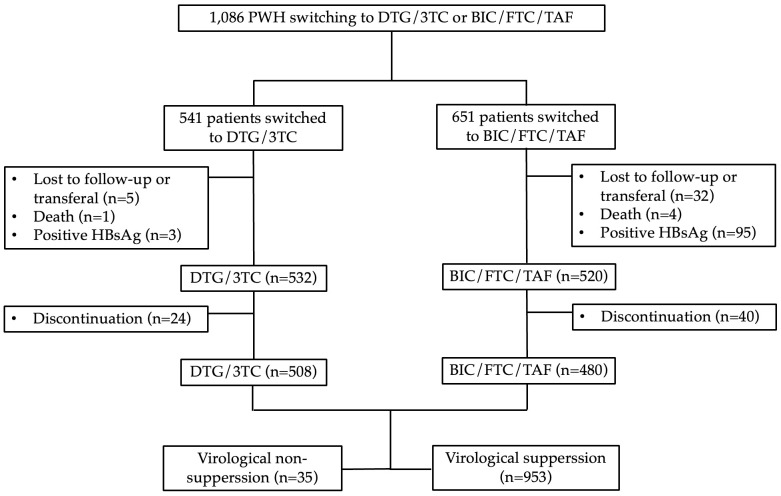
Flowchart for the inclusion of treatment-experienced patients. Abbreviation: PWH, people living with HIV; BIC/FTC/TAF, bictegravir/emtricitabine/tenofovir alafenamide; DTG/3TC, dolutegravir/lamivudine.

Between October 2019 and January 2023, we first screened 1,086 individuals and then included 988 individuals who switched their ART regimen to BIC/FTC/TAF or DTG/3TC, after excluding those who did not meet the inclusion criteria. (Fig 1) Among these, 93.4% were male, 73.0% were men who have sex with men, and 22.6% had a positive HCV antibody. Their median age (IQR) was 38 (32–46) years, and 152 (15.4%) were >50 years old. The median follow-up time since initiating ART was 5.2 (3.1–8.0) years. The mean CD4 counts was 607 cells/μL (434–796), and 34 (3.4%) had CD4 counts <200 cells/μL; 14 (1.4%) had HIV RNA levels >100,000 copies/mL, 77 (7.8%) had HIV RNA levels between 1000 and 100,000 copies/mL, 22 (2.2%) had LLV, and 875(88.6%) had VS at switch.

Moreover, 55 (5.6%) had the record of virological failure more than 2 times before switch. Out of these, 480 (48.6%) individuals switched their ART regimen to BIC/FTC/TAF, while 508 (51.4%) switched to DTG/3TC. VS at weeks 48 was achieved in 953 of 988 (96.5% [95% CI, 95.5%–97.4%]), and baseline characteristics of the individuals are summarized in Table 1.

**Table 1:** Baseline demographics and clinical characteristics in the study population.

	Overall (n = 988)	Virological non-suppression (n = 35)	Virological suppression (n = 953)	*p*
Age, y/o, median (IQR)	38 (32–46)	37 (33–40)	38 (32–46)	0.046
Male, n (%)	923 (93.4)	34 (97.1)	889 (93.3)	0.37
HCV Ab positive, n (%)	213 (22.6)	7 (20.0)	206 (22.4)	0.82
Route of transmission				
Men who have sex with men, n (%)	721 (73.0)	33 (94.3)	688 (72.4)	<0.01
Injection drug use, n (%)	168 (17.0)	1 (2.9)	167 (17.3)	0.02
Heterosexual contact, n (%)	80 (8.1)	1 (2.9)	79 (8.2)	0.35
Other, n (%)	19 (1.9)	0 (0)	19 (2.0)	0.39
CD4 counts at switch, cells/uL, median (IQR)	607 (434–796)	599 (386–809)	607 (435–795)	0.74
CD4 counts >500 cells/uL, n (%)	614 (62.1)	22 (62.9)	592 (621.5)	0.71
CD4 counts <200 cells/uL, n (%)	34 (3.4)	5 (14.3)	29 (3.0)	<0.01
HIV RNA level at switch				
<50 copies/mL, n (%)	875 (88.6)	18 (51.4)	857 (89.0)	<0.01
>100,000 copies/mL, n (%)	14 (1.4)	4 (11.4)	10 (1.0)	<0.01
Low level viremia^1^, n (%)	22 (2.2)	5 (14.3)	17 (1.8)	<0.01
Record of virological failure^2^, n (%)				
Never	840 (85.0)	21 (60.0)	819 (85.01)	<0.01
Once	148 (15.0)	14 (40.0)	134 (13.9)	<0.01
More than 2 times	55 (5.6)	11 (31.4)	44 (4.6)	<0.01
Previous ART regimen				0.74
Multiple tablet regimen, n (%)	49 (5.0)	3 (8.6)	46 (4.8)	
TDF/FTC/EFV, n (%)	60 (6.1)	2 (5.7)	58 (6.0)	
BIC/FTC/TAF, n (%)	11 (1.1)	0 (0)	11 (1.1)	
TDF/FTC/RPV, n (%)	14 (1.4)	1 (2.9)	13 (1.3)	
TAF/FTC/EVG/c, n (%)	330 (33.4)	8 (22.9)	322 (33.4)	
DTG/RPV, n (%)	89 (9.0)	2 (5.7)	87 (9.0)	
DTG/3TC/ABC, n (%)	414 (41.9)	18 (51.4)	396 (421.1)	
TAF/FTC/RPV, n (%)	21 (2.1)	2 (5.7)	19 (2.0)	
ART duration before switch, years, median (IQR)	5.2 (3.1–8.0)	5.0 (3.3–7.0)	5.0 (3.1–8.0)	0.62
ART regimen after switch				0.02
BIC/FTC/TAF, n (%)	480 (48.6)	24 (68.6)	456 (47.4)	
DTG/3TC, n (%)	508 (51.4)	11 (31.4)	497 (51.6)	

^1^Low-level viremia was characterized by HIV RNA levels between 50 to 1,000 copies/mL occurring more than twice consecutively in the previous year before the switch;

^2^virological failure was defined as a plasma HIV RNA levels exceeding 1,000 copies/mL.

Abbreviation: HCV, hepatitis C virus; ART, anti-retroviral therapy; TDF, Tenofovir fumarate; FTC, emtricitabine; EFV, efavirenz; BIC, bictegravir; TAF, tenofovir alafenamide; RPV, rilpivirine; EVG/c, elvitegravir/cobicistat; DTG, dolutegravir; ABC, abacavir.

### Pre-existing primary resistance-associated mutations

Before the switch, 252 (49.6%) PWH had data on RAMs in the DTG/3TC group, and 213 (44.4%) PWH had data in the BIC/FTC/TAF group. Pre-existing primary RAM to any drug class were identified in 24.2% (61/252) of patients in the DTG/3TC group and 31.5% (67/213) in the BIC/FTC/TAF group. The only significant difference in major RAM was M184V/I, with a prevalence of 7.0% in BIC/FTC/TAF compared to 2.4% in DTG/3TC, resulting in a difference of 4.6% (95% CI 1.6%-7.6%, p<0.01). Notably, one patient had K65R+M184V, and 2 patients had Q148H before switch in the DTG/3TC group. In the BIC/FTC/TAF group, three patients had K65R+M184V/I, and one patient had Q148H+G140S before switch (Table 2).

**Table 2:** Primary resistance associated mutations before the switch.

	DTG/3TC (n = 252)	BIC/FTC/TAF (n = 213)		DTG/3TC (n = 252)	BIC/FTC/TAF (n = 213)
**Primary** **NRTI-R**	20 (7.9%)	31 (14.6%)	**Primary** **NNRTI-R**	34 (13.5%)	33 (15.5%)
K65R/E/N	3 (1.2%)	3 (1.4%)	L100I	6 (2.4%)	3 (1.4%)
M184V/I*	6 (2.4%)	15 (7.0%)	K101E/P	4 (1.6%)	2 (0.9%)
L74V	1 (0.4%)	2 (0.9%)	K103N/S	7 (2.8%)	11 (5.2%)
Y115F	0	3 (1.4%)	V106A	8 (3.2%)	10 (4.7%)
T69NT	2 (0.8%)	9 (4.2%)	V108I	2 (0.8%)	1 (0.5%)
Any TAM^a^			E138A/G/K/Q	7 (2.8%)	5 (2.3%)
D67N	2 (0.8%)	5 (2.3%)	V179L	4 (1.6%)	5 (2.3%)
K70R	4 (1.6%)	3 (1.4%)	Y181C	3 (1.2%)	4 (1.9%)
L210W	0	1 (0.5%)	Y188L	1 (0.4%)	5 (2.3%)
T215F/Y	4 (1.6%)	3 (1.4%)	G190A/Q/S	3 (1.2%)	3 (1.4%)
K219E/N/Q/R	1 (0.4%)	2 (0.9%)	H221Y	1 (0.4%)	3 (1.4%)
			M230I	1 (0.4%)	0
**Primary** **PI-R**	5 (2.0%)	1 (0.5%)	**Primary** **INSTI-R**	2 (0.8%)	2 (0.9%)
M46I/L	3 (1.2%)	1 (0.5%)	T66I	0	1 (0.5%)
I54L	1 (0.4%)	0	G140S	0	1 (0.5%)
V82A/L	1 (0.4%)	0	Q148H	2 (0.8%)	1 (0.5%)

^a^ TAMs are M41L, D67N, K70R, L210W, T215F/Y, and K219E/N/Q/R in RT;

*p<0.05

Abbreviation: NRTI, nucleoside reverse transcriptase inhibitor; TAMs, thymidine analog mutations; NNRTI, non-nucleoside reverse transcriptase inhibitor; PI, protease inhibitor; INSTI, integrase strand transfer inhibitor; R, resistance.

Among PWH switching to DTG/3TC or BIC/FTC/TAF who had pre-existing K65R with or without M184V/I before the switch, all 3 out of 3 (100%) and 3 out of 3 (100%), respectively, had a plasma HIV RNA level of <50 copies/mL at time of switch and successfully maintained <50 copies/mL at week 48. It is worth noting that these 6 patients already achieved VS at baseline. Furthermore, among PWH with pre-existing M184V/I, 15 out of 21 (71.4%) patients had VS at the time of the switch. Among these, 5 out of 6 (83.3%) in the DTG/3TC group and 15 out of 15 (100%) in BIC/FTC/TAF group had an HIV RNA level of <50 copies/mL at week 48. Among the 6 patients with VNS at the time of switch, 6 out of 6 (100%) achieved VS. However, 1 out of 15 (6.7%) patients with VS at switch failed to maintain an HIV RNA level of <50 copies/mL at week 48. For patients with M184V/I, the median duration of previous VS before switch was 3.6 years (IQR: 2.5–5.5). Among individuals with Q148H genotype in the DTG/3TC group, two patients had HIV RNA levels viral loads of 72 and <50 copies/mL at the time of switch, respectively. In the BIC/FTC/TAF group, one patient with Q148H and G140S had an HIV RNA level of 13,700copies/mL at the time of switch. However, all three patients achieved VS by week 48. Moreover, among the 35 patients (3.5%) with VNS at week 48, no one was detected to have acquired drug resistance-associated mutations. Of the 35 patients, only 7 had HIV RNA levels >1000 copies/mL, which is sufficient for detecting RAM, while the remaining 28 had HIV RNA levels between 50 and 1000 copies/mL.

### Risk factors of virological non-suppression at week 48 after switch

As shown in Table 3, VNS were analyzed using a logistic regression model, which showed a Nagelkerke R Square of 0.218 and a Hosmer-Lemeshow test result of 0.582, indicating a moderate fit. After adjusting for baseline age, sex, route of transmission, primary RAM, CD4 cell counts, HIV RNA, low-level viremia, record of virological failure, and ART duration and regimens. The following factors before the switch were considered significantly: a record of virological failure ≥ 2 times (AOR 5.32, 95% CI 2.04–13.85), while an HIV viral load <50 copies/mL within the past three months before switch (AOR: 0.27, 95% CI 0.11–0.72) was identified as a protective factor.

**Table 3:** The analysis of risk factors of HIV RNA levels ≥ 50 copies/mL at week 48.

	Crude Odds Ratio (95% CI)	*p*	Adjusted Odds Ratio (95% CI)	*p*
Gender, male vs. female	2.51 (0.34–18.65)	0.37	1.23 (0.10–15.31)	0.87
Age (years), ≥50 vs. <50	0.88 (0.34–2.31)	0.80	0.99 (0.34–2.86)	0.99
Men who have sex with men, yes vs. no	4.25 (1.29–13.96)	0.02	4.19 (0.5–35.08)	0.19
Injection drug use, yes vs. no	0.28 (0.07–1.16)	0.08	0.49 (0.03–7.03)	0.60
Positive HCV antibody, yes vs. no	1.03 (0.46–2.30)	0.94	1.97 (0.73–5.30)	0.18
Primary NRTI-R, yes vs. no	3.19 (1.18–8.58)	0.02	2.48 (0.74–8.30)	0.14
Primary NNRTI-R, yes vs. no	1.77 (0.61–5.16)	0.30	0.82 (0.22–3.14)	0.78
CD4 count (cells/μL), <200 vs. ≥200	5.15 (1.87–14.20)	<0.01	1.39 (0.39–4.95)	0.61
HIV RNA level (copies/mL), <50 vs. ≥50	0.13 (0.06–0.25)	<0.01	0.27 (0.11–0.72)	<0.01
Low level viremia, yes vs. no	8.90 (3.09–26.67)	<0.01	2.26 (0.60–8.49)	0.23
Virological failure ≥2 times, yes vs. no	9.11 (4.21–19.70)	<0.01	5.32 (2.04–13.85)	<0.01
ART duration (years), ≥3 vs. <3	0.53 (0.26–1.06)	0.07	0.57 (0.26–1.23)	0.15
ART regimen, DTG/3TC vs. BIC/FTC/TAF	0.46 (0.23–0.92)	0.03	0.77 (0.35–1.70)	0.51

Abbreviation: NRTI, nucleoside reverse transcriptase inhibitor; NNRTI, non-nucleoside reverse transcriptase inhibitor; R, resistance; ART, anti-retroviral therapy.

At week 48, DTG/3TC was noninferior to BIC/FTC/TAF in achieving HIV RNA <50 copies/mL (-10% noninferiority margin) in the per-protocol analysis (proportion of responders, 98.2% vs 95.0%, respectively; adjusted treatment difference = 3.2%, 95%CI, 0.7% - 5.3%]). Moreover, the incidence rate of developing LLV was 3.5 per 100 person-years of follow-up (PYFU) in the BIC/FTC/TAF group and 2.2 per 100 PYFU in the DTG/3TC group [incidence rate ratio (IRR) = 1.64, 95%CI, 0.77–3.49, *p* = 0.09].

### Immunological response and clinical outcomes

**Fig 2:**
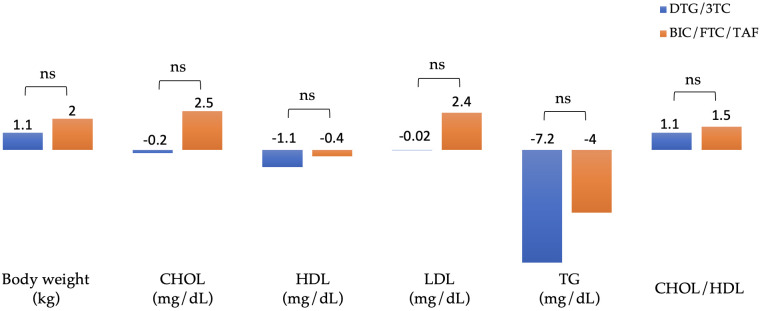
Changes in body weight and blood lipid from baseline to week 48. Footnote: The mean difference of body weight (n = 380), CHOL (n = 705), HDL (n = 683), LDL (n = 685), TG (n = 701) and CHOL/HDL (n = 646) between groups were analyzed in the PP-E analysis. Abbreviation: CHOL, total cholesterol; HDL, high density lipoprotein; LDL, low density lipoprotein; TG, triglyceride; PP-E, per-protocol exposed *p <0.05; ns, not significant.

The mean (standard deviation [SD]) change from baseline to week 48 in CD4 lymphocyte cell count was 63 cells/μL (± 221) in the DTG/3TC group and 51 cells/μL (± 209) in the BIC/FTC/TAF group (*p* = 0.39). Fig 2 summarizes the changes in body weight and lipid profiles of patients after 48 weeks of switching to DTG/3TC and BIC/FTC/TAF. The absolute mean (SD) weight at baseline versus week 48 was 72.4 (± 13.7) vs. 73.4 (± 13.6) kg in the DTG/3TC group (n = 209) and 70.3 (± 11.9) vs. 72.3 (± 12.5) kg in the BIC/FTC/TAF group (n = 171), respectively. In the BIC/FTC/TAF group, body weight increased by 2.0 kg, and in the DTG/3TC group, body weight increased by 1.1 kg (adjusted difference, 0.9 kg; 95% CI, 0.4–1.4; *p* = 0.053).

The absolute mean total cholesterol (CHOL), high-density lipoprotein (HDL), low-density lipoprotein (LDL) and mean triglyceride (TG) at baseline were 182 (± 33), 48 (± 13), 121 (± 31) and 142 (± 109) mg/dL in the DTG/3TC group, and 179 (± 34), 48 (± 13), 115 (± 29) and 150 (± 100) mg/dL in the BIC/FTC/TAF group, respectively. The absolute mean CHOL/HDL ratio at baseline was 4.0 (± 1.1) in the DTG/3TC group (n = 376), and 3.9 (± 1) in the BIC/FTC/TAF group (n = 270). Blood lipid parameters did not differ substantially from baseline to week 48, including total cholesterol, high-density lipoprotein, low-density lipoprotein, and triglyceride.

### Adverse events and causes leading to discontinuation

**Fig 3:**
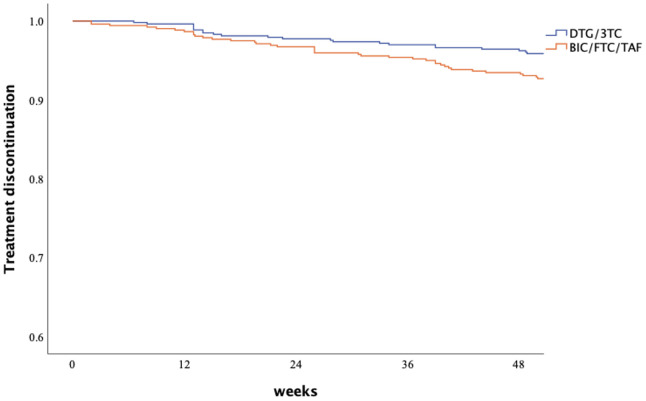
Kaplan–Meier plot of accumulative treatment discontinuation for DTG/3TC and BIC/FTC/TAF.

Through week 48, the proportion of patients reporting drug-related adverse events or causes leading to discontinuation was 24 (4.5%) in the DTG/3TC group and 40 (7.7%) in the BIC/FTC/TAF group. The most common adverse events (AEs) in the DTG/3TC group were pruritus (0.9%), insomnia (0.8%), headache/dizziness (0.4%), increased weight (0.4%), and renal function deterioration (0.4%). The most common AEs leading to discontinuation in the BIC/FTC/TAF group were increased weight or obesity (1.5%), insomnia (0.8%), nausea/diarrhea (0.8%) and low-level viremia (0.8%). Treatment discontinuation rates did not significantly differ between the two regimens (Table 4) Fig 3. illustrates the Kaplan–Meier plot displaying the cumulative discontinuation rate, and the hazard ratio for discontinuation of BIC/FTC/TAF was 1.65 (95% CI, 0.99–2.74, *p* = 0.055).

**Table 4:** The adverse effects and causes leading to discontinuation in the study.

	DTG/3TC (n = 532)	BIC/FTC/TAF (n = 520)
all events of discontinuation, n (%)	24 (4.5)	40 (7.7)
renal function deterioration, n (%)	2 (0.4)	3 (0.6)
numbness, n (%)	1 (0.2)	1 (0.2)
fatigue, n (%)	0	2 (0.4)
headache/dizziness, n (%)	2 (0.4)	1 (0.2)
anxiety, n (%)	1 (0.2)	0
insomnia, n (%)	4 (0.8)	4 (0.8)
pruritus, n (%)	5 (0.9)	2 (0.4)
nausea/diarrhea, n (%)	1 (0.2)	4 (0.8)
blood sugar poor control, n (%)	0	2 (0.4)
hyperlipidemia, n (%)	0	3 (0.6)
virological failure, n (%)	1 (0.2)	2 (0.4)
low level viremia, n (%)	1 (0.2)	4 (0.8)
drug drug interaction, n (%)	0	1 (0.2)
increased weight or obesity, n (%)	2 (0.4)	8 (1.5)
completed LTBI treatment, then shift back to previous ART^1^, n (%)	2 (0.4)	1 (0.2)

^1^After completing LTBI treatment, some patients preferred to switch back to their previous ART regimen.

Abbreviation: LTBI, latent tuberculosis infection

## Discussion

In this real-world study involving a switch to BIC/FTC/TAF or DTG/3TC in 988 ART-experienced PWH, we observed high proportions of suppressed HIV RNA level at week 48. However, the occurrence of HIV RNA levels greater than 50 copies/mL at week 48 after switch was associated with a history of virological failure more than 2 times before the switch. Furthermore, having suppressed HIV RNA level before the switch was associated with a lower risk of HIV RNA levels greater than 50 copies/mL at week 48, even among PWH with pre-existing NRTI resistance.

BIC/FTC/TAF and DTG/3TC are efficacious and well-tolerated INSTI-based regimens for both ART-naïve [5, 13, 14] and ART-experienced people living with HIV [4, 6, 15–19]. They are recommended as first-line ART options in several international HIV treatment. TANGO and SALSA clinical trials revealed that DTG/3TC maintained virological suppression similarly to a TDF or TAF-based three-drug regimen. Our cohort also showed switching to DTG/3TC maintained similar virological suppression as switching to BIC/FTC/TAF at week 48. The proportion of suppressed HIV RNA level was 98% (DTG/3TC) and 98% (BIC/FTC/TAF) in the subgroup of HIV RNA level <50 copies/mL at the time of switch. Moreover, the interval change from the time of switch in CD4 lymphocyte cell count was not significantly different between the DTG/3TC and BIC/FTC/TAF groups (63 vs. 51 cells/μL, *p* = 0.39), and the finding was similar to TANGO and SALSA studies.

Clinical trials may exhibit the weakness of lacking diversity, with enrolled participants not reflecting the demographics of the intended patient population, thereby limiting the interpretation of published outcomes. On the other hand, retrospective observational cohort studies face the weakness of selection bias. Participants in real-world studies may not be randomly assigned, leading to the potential for a disproportion between groups. This can affect the generalizability of the findings to the broader population [20]. Therefore, we conducted a logistic regression model to identify the risk factors for VNS at week 48. While the TANGO and SALSA studies only enrolled participants limited to VS for more than 6 months, our real-world study included those with VNS at the switch (n = 113, 11.4%). We then proceeded to identify the risk factors in a broader context.

LLV with HIV RNA levels between 50 and 1000 copies/mL could lead to subsequent virological failure (HIV RNA levels >1000 copies/mL) before the era of second-generation integrase strand transfer inhibitor-containing regimens as first-line ART [8, 21, 22]. Chen GJ et al. showed that the risks of developing LLV were similarly low between PWH switched to bictegravir-based (6.2 per 100 person-years) and dolutegravir-based regimens (3.8 per 100 person-years) [IRR = 1.63, 95%CI, 0.90–2.95] [23]. Moreover, the incidences of LLV were 13.2 and 7.0 per 100 person-years of follow-up in the dolutegravir and PI group, respectively [IRR = 1.90, 95% CI, 0.99–3.62] [24]. The results were consistent with our study, which demonstrated that the incidence rate of developing LLV was 3.5 per 100 person-years of follow-up in the BIC/FTC/TAF group and 2.2 per 100 PYFU in the DTG/3TC group [IRR = 1.64, 95%CI, 0.77–3.49].

Some studies confirmed that DTG/3TC and BIC/FTC/TAF were effective, regardless of the existence of the M184V mutation, among suppressed PWH. Andreatta K. et al revealed that at week 48, 98% (561/570) of all BIC/FTC/TAF-treated participants versus 98% (213/217) with pre-existing resistance and 96% (52/54) with archived M184V/I had HIV RNA level <50 copies/mL. No BIC/FTC/TAF-treated participants developed resistance-associated mutations (RAMs) to study drugs [16]. Santoro MM. et al showed that the probability of virological failure and blips in patients switching to DTG/3TC was very low (7.8% and 6.9%) after 3 years of treatment regardless of M184V [25]. Our study found that among PWH switching to DTG/3TC or BIC/FTC/TAF with pre-existing K65R, with or without M184V/I, all seven individuals (100%) had HIV RNA levels less than 50 copies/mL at the time of the switch, and they maintained HIV RNA levels below 50 copies/mL at week 48. However, the effect of a short duration (median of 3.6 years, IQR: 2.5–5.5) of previous virological suppression in patients with M184V/I on DTG/3TC or BIC/FTC/TAF response remains unclear. Therefore, a clinical trial that examines the duration of virological suppression before the switch is warranted.

The mean increase in body weight from the time of switch to week 48 was 2.0 kg in the BIC/FTC/TAF group and 1.1 kg in the DTG/3TC group (adjusted difference, 0.9 kg; 95% CI, 0.4–1.4; *p* = 0.053). This difference in weight gain between groups may be partly explained by patients in the BIC/FTC/TAF group switching from regimens (TDF/FTC/EFV and TDF/FTC/RPV) known to be associated with weight gain suppression (BIC/FTC/TAF vs DTG/3TC; 11% vs 3%; mean difference, 8%; 95% CI, 5%–11%; *p* <0.01) [26, 27]. Moreover, overall adverse effects were generally similar between groups, but increased weight or obesity was the leading cause of discontinuation in patients switching to a regimen of BIC/FTC/TAF than those switching to DTG/3TC. Despite the 0.9 kg difference in weight increase, lipid profile was generally unchanged from baseline across both groups.

Our study has several limitations, and the results should be carefully interpreted. First, this was a retrospective study with unbalanced the time of switch characteristics between the two groups, and some of the discontinuations were made by the treating physicians. Therefore, we used a logistic regression model for multivariate analyses to adjust for possible confounding factors or bias. Second, the small number of VNS at week 48 limited our statistical power when constructing a multivariate model to identify potential risk factors. Third, the included people living with HIV were mainly taking single-tablet regimens before switching to DTG/3TC or BIC/FTC/TAF, and its generalizability was limited to PWH who took multiple-tablet regimens. Fourth, around 50% of all the included PWH had data on resistance-associated mutations before the switch because antiretroviral resistance testing was not routinely available in PWH who initiated or switched ART in Taiwan. Fifth, the small number of patients (6%) with a record of virological failure more than two times before switching can hinder the probability of developing a robust model with sufficient power to achieve accurate predictions. Additionally, with only 35 patients experiencing VNS, the ability to include multiple adjustment variables in the model is constrained, and this restriction can impact the comprehensiveness and predictive accuracy of the model. Sixth, the study was conducted through a per-protocol analysis and not by intention-to-treat. Finally, this was a single-center study that lasted for only 48 weeks, limiting the ability to extrapolate.

## Conclusions

In conclusion, our study suggests that the effectiveness and safety profiles of switching to DTG/3TC and BIC/FTC/TAF were comparable in treatment-experienced adults with HIV, especially among those with VS at the time of switch. However, frequent virological failure before the switch might impact the benefits of these regimens in the short term of follow-up.

## Supporting information

S1 Raw data (XLSX)

## References

[1] Panel on Antiretroviral Guidelines for Adults and Adolescents. Guidelines for the use of antiretroviral agents in adults and adolescents with HIV. 2023. https://clinicalinfo.hiv.gov/sites/default/files/guidelines/documents/adult-adolescent-arv/guidelines-adult-adolescent-arv.pdf. Accessed 06 December 2023.

[2] European AIDS Clinical Society. EACS guidelines version 12.0. October 2023. https://www.eacsociety.org/media/guidelines-12.0.pdf Accessed October 2023

[3] Gandhi RT, Bedimo R, Hoy JF, Landovitz RJ, Smith DM, Eaton EF, et al. Antiretroviral drugs for treatment and pre vention of HIV infection in adults: 2022 recommendations of the International Antiviral Society—USA panel. JAMA. 2023; 329: 63–84. doi: 10.1001/jama.2022.22246 36454551

[4] Wyk JV, Ajana F, Bisshop F, Wit SD, Osiyemi O, Sogorb J.P, et al. Efficacy and safety of switching to dolutegravir/lamivudine fixed-dose 2-drug regimen vs continuing a tenofovir alafenamide—based 3- or 4-drug regimen for maintenance of virologic suppression in adults living with human immunodeficiency virus type 1: phase 3, randomized, noninferiority TANGO study. Clin Infect Dis. 2020; 71: 1920–9. doi: 10.1093/cid/ciz1243 31905383 PMC7643745

[5] Cahn P, Madero JS, Arribas JR, Antinori A, Ortiz R, Clarke AE, et al. Dolutegravir plus lamivudine versus dolutegravir plus tenofovir disoproxil fumarate and emtricitabine in antiretroviral-naive adults with HIV-1 infection (GEMINI-1 and GEMINI-2): week 48 results from two multicentre, double-blind, randomised, non-inferiority, phase 3 trials. Lancet. 2019; 393: 143–155. doi: 10.1016/S0140-6736(18)32462-0 30420123

[6] Llibre JM, Brites C, Cheng CY, Osiyemi O, Galera C, Hocqueloux L, et al. Efficacy and Safety of Switching to the 2-Drug Regimen Dolutegravir/Lamivudine Versus Continuing a 3- or 4-Drug Regimen for Maintaining Virologic Suppression in Adults Living With Human Immunodeficiency Virus 1 (HIV-1): Week 48 Results From the Phase 3, Noninferiority SALSA Randomized Trial. Clin Infect Dis. 2023; 76: 720–729. doi: 10.1093/cid/ciac130 35235656 PMC10021070

[7] Bernal E, Gómez JM, Jarrín I, Cano A, Muñoz A, Alcaraz A, et al. Low-Level Viremia Is Associated With Clinical Progression in HIV-Infected Patients Receiving Antiretroviral Treatment. J Acquir Immune Defic Syndr. 2018; 78: 329–337. doi: 10.1097/QAI.0000000000001678 29543636

[8] Joya C, Won SH, Schofield C, Lalani T, Maves RC, Kronmann K, et al. Persistent Low-level Viremia While on Antiretroviral Therapy Is an Independent Risk Factor for Virologic Failure. Clin Infect Dis. 2019; 69: 2145–2152. doi: 10.1093/cid/ciz129 30785191 PMC6880328

[9] Zhang T, Ding H, An M, Wang X, Tian W, Zhao B, et al. Factors associated with high-risk low-level viremia leading to virologic failure: 16-year retrospective study of a Chinese antiretroviral therapy cohort. BMC Infect Dis. 2020; 20: 147. doi: 10.1186/s12879-020-4837-y 32066392 PMC7026956

[10] Mesic A, Spina A, Mar HT, Thit P, Decroo T, Lenglet A, et al. Predictors of virological failure among people living with HIV receiving first line antiretroviral treatment in Myanmar: retrospective cohort analysis. AIDS Res Ther. 2021; 18: 16. doi: 10.1186/s12981-021-00336-0 33882962 PMC8059266

[11] Wensing AM, Calvez V, Ceccherini-Silberstein F, Charpentier C, Günthard HF, Paredes R, et al. 2019 update of the drug resistance mutations in HIV-1. Top Antivir Med. 2019; 27: 111–121. 31634862 PMC6892618

[12] HIVdb program of the Stanford University HIV Drug Resistance Database, updated Oct 25 2022. https://cms.hivdb.org/prod/downloads/resistance-mutation-handout/resistance-mutation-handout.pdf

[13] Gallant J, Lazzarin A, Mills A, Orkin C, Podzamczer D, Tebas P, et al. Bictegravir, emtricitabine, and tenofovir alafenamide versus dolutegravir, abacavir, and lamivudine for initial treatment of HIV-1 infection (GS-US-380-1489): a double-blind, multicentre, phase 3, randomised controlled non-inferiority trial. Lancet. 2017; 390: 2063–2072. doi: 10.1016/S0140-6736(17)32299-7 28867497

[14] Sax PE, Pozniak A, Montes ML, Koenig E, DeJesus E, Stellbrink HJ, et al. Coformulated bictegravir, emtricitabine, and tenofovir alafenamide versus dolutegravir with emtricitabine and tenofovir alafenamide, for initial treatment of HIV-1 infection (GS-US-380-1490): a randomised, double-blind, multicentre, phase 3, non-inferiority trial. Lancet. 2017; 390: 2073–2082. doi: 10.1016/S0140-6736(17)32340-1 28867499

[15] Sax PE, Rockstroh JK, Luetkemeyer AF, Yazdanpanah Y, Ward D, Trottier B, et al. GS-US-380–4030 Investigators. Switching to Bictegravir, Emtricitabine, and Tenofovir Alafenamide in Virologically Suppressed Adults With Human Immunodeficiency Virus. Clin Infect Dis. 2021; 73: e485–e493. 32668455 10.1093/cid/ciaa988 PMC8282313

[16] Andreatta K, Willkom M, Martin R, Chang S, Wei L, Liu H, et al. Switching to bictegravir/emtricitabine/tenofovir alafenamide maintained HIV-1 RNA suppression in participants with archived antiretroviral resistance including M184V/I. J Antimicrob Chemother. 2019; 74: 3555–3564. doi: 10.1093/jac/dkz347 31430369 PMC6857193

[17] Daar ES, DeJesus E, Ruane P, Crofoot G, Oguchi G, Creticos C, et al. Efficacy and safety of switching to fixed-dose bictegravir, emtricitabine, and tenofovir alafenamide from boosted protease inhibitor-based regimens in virologically suppressed adults with HIV-1: 48 week results of a randomised, open-label, multicentre, phase 3, non-inferiority trial. Lancet HIV. 2018; 5: e347–e356. doi: 10.1016/S2352-3018(18)30091-2 29925490

[18] Molina. JM, Ward D, Brar I, Mills A, Stellbrink HJ, López-Cortés L, et al. Switching to fixed-dose bictegravir, emtricitabine, and tenofovir alafenamide from dolutegravir plus abacavir and lamivudine in virologically suppressed adults with HIV-1: 48 week results of a randomised, double-blind, multicentre, active-controlled, phase 3, non-inferiority trial. Lancet HIV. 2018; 5: e357–e365. doi: 10.1016/S2352-3018(18)30092-4 29925489

[19] Mendoza I, Lázaro A, Espinosa A, Sánchez L, Horta AM, Torralba M. Effectiveness, durability and safety of dolutegravir and lamivudine versus bictegravir, emtricitabine and tenofovir alafenamide in a real-world cohort of HIV-infected adults. PLoS One. 2023; 18(9): e0291480. doi: 10.1371/journal.pone.0291480 37773939 PMC10540944

[20] Rubin E. Striving for diversity in research studies. N Engl J Med. 2021; 385: 1429–30. doi: 10.1056/NEJMe2114651 34516052

[21] Navarro J, Caballero E, Curran A, Burgos J, Ocaña I, Falcó V, et al. Impact of low-level viraemia on virological failure in HIV-1- infected patients with stable antiretroviral treatment. Antivir Ther. 2016; 21: 345–52. doi: 10.3851/IMP3023 26756461

[22] Hermans LE, Moorhouse M, Carmona S, Grobbee DE, Hofstra LM, Richman DD, et al. Effect of HIV-1 low-level viraemia during antiretroviral therapy on treatment outcomes in WHO-guided South African treatment programmes: a multicentre cohort study. Lancet Infect Dis. 2018; 18: 188–97. doi: 10.1016/S1473-3099(17)30681-3 29158101

[23] Chen GJ, Sun HY, Chen LY, Hsieh SM, Sheng WH, Liu WD, et al. Low-level viraemia and virologic failure among people living with HIV who received maintenance therapy with co-formulated bictegravir, emtricitabine and tenofovir alafenamide versus dolutegravir-based regimens. Int J Antimicrob Agents. 2022; 60: 106631. doi: 10.1016/j.ijantimicag.2022.106631 35787920

[24] Chen GJ, Sun HY, Chang SY, Cheng A, Huang YS, Huang SH, et al. Incidence and impact of low-level viremia among people living with HIV who received protease inhibitor- or dolutegravir-based antiretroviral therapy. Int J Infect Dis. 2021; 105: 147–151. doi: 10.1016/j.ijid.2021.02.045 33592339

[25] Santoro MM, Armenia D, Teyssou E, Santos JR, Charpentier C, Lambert-Niclot S, et al. Virological efficacy of switch to DTG plus 3TC in a retrospective observational cohort of suppressed HIV-1 patients with or without past M184V: the LAMRES study. J Glob Antimicrob Resist. 2022; 31: 52–62. doi: 10.1016/j.jgar.2022.07.022 35948240

[26] Sax PE, Erlandson KM, Lake JE, Mccomsey GA, Orkin C, Esser S, et al. Weight gain following initiation of antiretroviral therapy: risk factors in randomized comparative clinical trials. Clin Infect Dis. 2020; 71: 1379–89. doi: 10.1093/cid/ciz999 31606734 PMC7486849

[27] Huang SH, Huang WC, Lin SW, Chuang YC, Sun HY, Chang SY, et al. Impact of efavirenz mid-dose plasma concentration on long-term weight change among virologically suppressed people living with HIV. J Acquir Immune Defic Syndr. 2021; 87: 834–41. doi: 10.1097/QAI.0000000000002650 33587507

