# Supplementary material for: Assessment of risk factors for virological nonsuppression following switch to dolutegravir and lamivudine, or bictegravir, emtricitabine, and tenofovir alafenamide fumarate in a real-world cohort of treatment-experienced adults living with HIV
Source: PLoS One. 2024 Nov 20;19(11):e0314003. doi: 10.1371/journal.pone.0314003 (PMC11578452; doi:10.1371/journal.pone.0314003)
Supplement: Attachment — Submitted filename: responses to reviewers.docx [file pone.0314003.s002.docx]

Dear Editor,

We would like to thank you and the reviewers for giving us the opportunity to improve our manuscript entitled “**Assessment of risk factors for virological nonsuppression following switch to dolutegravir and lamivudine, or bictegravir, emtricitabine, and tenofovir alafenamide fumarate in a real-world cohort of treatment-experienced adults living with HIV**”. Our point-to-point response to the reviewers' comments and queries are listed below.

1. Please ensure that your manuscript meets PLOS ONE's style requirements, including those for file naming.

Reply: We have revised the manuscript and included the requirements as attachments.

2. We noticed you have some minor occurrence of overlapping text with the following previous publication(s), which needs to be addressed:

Reply: We cited these 2 articles in reference 16 and 22, and the resistance mutation were analyzed in these 2 articles and our study, but we believed the outcome is slightly different.

3. We note that your Data Availability Statement is currently as follows: All relevant data are within the manuscript and its Supporting Information files.

Reply: We have uploaded all raw data as Supporting Information files.

4. Please include your tables as part of your main manuscript and remove the individual files. Please note that supplementary tables (should remain/ be uploaded) as separate ""supporting information"" files.

Reply: We have revised the manuscript and included the requirements as attachments.

Review Comments to the Author:

Reviewer 1:

Major comments:
1. Introduction:
The authors stated that the study aimed to explore the risk factors associated with virological non-suppression following a 48-week period of secondary INSTI-based ART. However, it's important to note that secondary INSTI-based ART includes more than just BIC/FTC/TAF and DTG/3TC.

Reply: Thank you for your important comment. However, BIC/FTC/TAF and DTG/3TC were prescribed dominantly at our hospital, while the remaining secondary INSTI-based ART prescriptions were less than 1%.

2. Methods: The study included PWH who were switched to either BIC/FTC/TAF or DTG/3TC but excluded those who discontinued ART, were lost to follow-up, or passed away from the final analyses. These exclusion criteria should be explicitly stated in the study's methodology. Moreover, PWH who discontinued ART, were lost to follow-up, or passed away could potentially have experience virological non-suppression. Excluding these individuals may introduce bias into the analysis.

Reply: Your idea is very important. We added a third exclusion criterion for those who discontinued ART, were lost to follow-up, or passed away. Additionally, our study is a per-protocol analysis, and all included patients should take BIC/FTC/TAF or DTG/3TC for a 48-week period.

3. Results:

(1) In the methodology, the authors indicated that the study was conducted from March 2019 to January 2022, with individuals followed up until January 2023. However, in the results, individuals were screened for inclusion between October 2019 and January 2023.

Reply: It’s from October 2019 and January 2023. We revised our Methods in page 4.
(2) A logistic regression model was used to clarify the risk factors associated with virological non-suppression. However, the authors included certain variables in the model that might have collinearity, such as a PVL >100,000 cp/mL, a history of virological failure ≥2 times, and PVL <50 cp/mL within the past 3 months before switch. It's crucial to inquire whether the authors assessed collinearity beforehand.

Reply: Thank for your valuable idea. We deleted 2 variables of PVL>100,000 copies/ml and HBsAg, because the other reviewer also suggested that positive HBsAg PLWH should be excluded, because only 3 patients with positive HBsAg was switched to DTG/3TC, but 95 patients were switched to BIC/FTC/TAF disproportionally.

(3) The authors concluded that the effectiveness of DTG/3TC and BIC/FTC/TAF was comparable based on the findings from the multivariable analysis. However, it's still essential to compare effectiveness outcomes, which included virological suppression, low-level viremia, viral blip, and virological failure, between DTG/3TC and BIC/FTC/TAF.

Reply: At week 48, DTG/3TC was noninferior to BIC/FTC/TAF in achieving HIV RNA < 50 copies/mL (-10% noninferiority margin) in the per-protocol analysis (proportion of responders, 98.2% vs 95.0%, respectively; adjusted treatment difference [95% CI], 3.2% [0.7& to 5.3%]).

(4) The authors mentioned that no individual was detected to have acquired drug RAMs during follow-up. How many individuals had viremia levels sufficient for detecting RAMs?

Reply: Only 7 individuals had HIV RNA> 1000 copies/mL which is sufficient for detecting RAM, and the rest (n=28) had HIV RNA between 50 to 1000 copies/mL.

(5) Since the tables are not included in the manuscript, I'm having difficulty reviewing the accuracy of the results.

Reply: Tables were attached in supplement files, and we revised the tables to our manuscript.

Minor comments:
1. Abstract:
(1) Line 8: A total of 1086 patients were included, 44 patients (4%) with VS at week 48.
-> Please check the number of patients with virological suppression (VS).

Reply: A total of 988 patients were included, 35 patients (3.5%) with VNS at week 48.
(2) In line with the study title, the conclusion should emphasize the impact of various ART regimens rather than focusing on subsequent RAMs.

Reply: In conclusion, DTG/3TC and BIC/FTC/TAF demonstrated good effectiveness in a real-world cohort, but frequent virological failure before the switch might impact the benefits of these regimens in the short-term follow-up.

2. Please standardize the terminology for HIV-1 RNA levels throughout the manuscript. Currently, different terms are used interchangeably, including HIV-1 RNA levels, HIV-VL, plasma viral load, etc.

Reply: Thank for your advice, and we unify this term as HIV RNA.

3. Please ensure that the full names of abbreviations are written out only once, when they first appear in the text.

Reply: Sure, we revise the abbreviations written out only once.

4. Introduction:
Paragraph 3: Most studies predominantly included treatment-naïve or virologically suppressed patients,
-> Please specify the types of ART studied in the mentioned studies.

Reply: We only included treatment-experienced patients who were either virologically suppressed or non-suppressed.

5. Methods:
Considering the correlation between positive HBsAg and virological non-suppression, and the decision not to switch PWH coinfected with HBV to DTG/3TC regimen, do you think positive HBsAg should be listed as an exclusion criterion?

Reply: Thank for your valuable idea. We deleted 2 variables of PVL>100,000 copies/ml and HBsAg, because the other reviewer also suggested that positive HBsAg PLWH should be excluded, because only 3 patients with positive HBsAg was switched to DTG/3TC, but 95 patients were switched to BIC/FTC/TAF disproportionally.

6. Results:
(1) Did those 44 patients with detectable viral loads at week 48 continue to use DTG/3TC and BIC/FTC/TAF?

Reply: Yes, 8 over 11 and 18 over 24 continue to use DTG/3TC or BIC/FTC/TAF, because most of them had blips or LLV, and the physicians and nurses boosted their adherence and encourage them to keep HIV treatment.
(2) Fig 2. Changes in body weight and blood lipid from baseline to week 48 in the PP-E analysis.
-> What does PP-E analysis stand for?

Reply: Per-Protocol-Exposed analysis

7. Discussion:
(1) Paragraph 3: "TANGO" should be capitalized.

Reply: thank you, we revised.
(2) Paragraph 3: Our real-world study included those with detectable viral loads at the switch (13.2%).
-> However, the results showed that 19 (2%) had PVL ≥100,000 cp/mL and 26 (2%) had LLV at the switch.

Reply: Exclusion criteria included positive HBsAg, therefore total included patients were revised as 988, and 875 patients with HIV RNA <50 copies/mL at switch; hence, 113 patients (11.3%) with detectable viral loads at switch.

(3) Paragraph 4: which demonstrated that the incidence rate of developing LLV was 4.2 per 100 person-years of follow-up in the BIC/FTC/TAF group and 2.3 per 100 PYFU in the DTG/3TC group [incidence rate ratio = 1.83; p = 0.09].
-> The data are not presented in the results part. Additionally, to compare incidences between studies, the authors should briefly list some data from previous studies.

Reply: Thank for your advice, and we revised my mauscript in “result” and “discussion”.

Chen GJ et al. showed that the risks of developing LLV were similarly low between PWH switched to bictegravir-based (6.2 per 100 person-years) and dolutegravir-based regimens (3.8 per 100 person-years) [IRR = 1.63, 95%CI, 0.90-2.95] [22]. Moreover, the incidences of LLV were 13.2 and 7.0 per 100 person-years of follow-up in the dolutegravir and PI group, respectively [IRR = 1.90, 95% CI, 0.99–3.62] [23]. The results were consistent with our study, which demonstrated that the incidence rate of developing LLV was 3.5 per 100 person-years of follow-up in the BIC/FTC/TAF group and 2.2 per 100 PYFU in the DTG/3TC group [IRR = 1.64, 95%CI, 0.77-3.49].

Reviewer 2:

Abstract:

VS: Acronyms must be defined. I understand that 44 patients (4%) were virological non suppressors. (VNS) A switch should be distinguished from undetectability vs. virological failure or treatment suspension and restart with a new regimen.
No significant difference was found between the DTG/3TC and BIC/FTC/TAF regimens (AOR: 1.32, 95% CI 0.59–2.94) (it is not known in the abstract which the OR is favorable towards). That is, using BIC/FTC/TAF multiplies the risk of failure compared to DTG/3TC by 1.32 (with DTG/3TC being the reference) or using DTG/3TC multiplies the risk of failure compared to BIC/FTC/TAF by 1.32
Reply: Positive HBsAg patients were excluded, therefore we analyzed the effectiveness of DTG/3TC and BIC/FTC/TAF. Totally, DTG/3TC was noninferior to BIC/FTC/TAF in achieving HIV RNA < 50 copies/mL (-10% noninferiority margin) in the per-protocol analysis (proportion of responders, 98.2% vs 95.0%, respectively; adjusted treatment difference [95% CI], 3.2% [0.7% to 5.3%]). However, among virologically suppressed patients, the effectiveness of DTG/3TC and BIC/FTC/TAF was 97.7% and 98.0%, respectively; adjusted treatment difference [95% CI], -0.3% [-1.9% to 1.3%]).

Introduction

The primary objective of this study is to investigate the risk factors associated with virological nonsuppression outcome with secondary INSTI-based ART after a 48-week period. The objective must be defined the population in which the risk factors for virological non-suppression will be analyzed (Patients in Switch). Do it in PICO format. Population (Switch), intervention will change to DTG/3TC (intervention) or BIC/FTC/TAF (comparison) and result (Virological non suppression)

Reply: The primary objective of this study is to investigate the risk factors associated with virological nonsuppression outcome with switching to DTG/3TC or BIC/FTC/TAF after a 48-week period among treatment-experienced patients who were either virologically suppressed or non-suppressed.

Material and methods.
In exclusion criteria..., were pregnant women excluded? Were patients with HBsAg+ not excluded? If not, Were any of them assigned to DTG/3TC?

Reply: Thank for your valuable idea. We deleted 2 variables of PVL>100,000 copies/ml and HBsAg, because the other reviewer also suggested that positive HBsAg PLWH should be excluded, because only 3 patients with positive HBsAg was switched to DTG/3TC, but 95 patients were switched to BIC/FTC/TAF disproportionally.

…with “virological non-suppression” (This terminology should be consistent throughout the manuscript)

Reply: thank you, we revised.

In the analysis it must be clear if the end point is through a “snapshot” at week 48 (regardless of what happens up to week 48 or if a confirmed virological failure is performed in a second sample if there is one).

Reply: Yes, it’s snapshot, per protocol analysis.

The authors comment: “Virological non-suppression included: LLV, viral blip, and VF”. In the case of a patient with a

VL of 75 copies/ml at w 48 and having previously VL < 50 copies at all times, would it be defined as VF without having analyzed a second VL to know if it is a blip?
Reply: Yes, it is defined as VNS, because HIV RNA can be measured only 4 times annually according to Taiwan CDC HIV guidelines.


Statistic analysis.
Why were parametric tests not carried out instead of non-parametric ones? You had samples of more than 30 subjects in each group. Was a paired data study ever carried out? (McNemar and Wilcoxon? Were they necessary?
In logistic regression models, it would be advisable to add model calibration using the Hosmer-Lemeshow test and Nagelkerke's R2 to assess the proportion of VNS that is explained by the independent variables.

Reply: Exactly, we performed non-parametric tests, because the data significantly deviate from normality. A paired data study is not ever carried, so we deleted this sentence. Thank for your advice, and we add the Hosmer-Lemeshow test and Nagelkerke's R2 to assess the proportion of VNS that is explained by the independent variables.

…”Furthermore, 62 (6%) had the record of virological failure more than 2 times before switching”. (There are few patients which can hinder the probability of having a model with sufficient power to achieve accurate predictions (with narrow 95% CI)
Failure was seen in 46 patients, which limits models preferably with no more than 5 adjustment variables...
Just comment in limitations

Reply: Fifth, the small number of patients (6%) with a record of virological failure more than two times before switching can hinder the probability of developing a robust model with sufficient power to achieve accurate predictions. Additionally, with only 35 patients experiencing VNS, the ability to include multiple adjustment variables in the model is constrained, and this restriction can impact the comprehensiveness and predictive accuracy of the model.

Results:
Do we know the percentage with RNA+ in those with HCV+ serology?

Reply: Sorry, we didn’t check the viremic status of patients with positive HCV antibody.

…Their mean age (SD) was 39.8 (± 9.9)… perhaps better median (p25-p75) years, and 173 (16%) were > 50 years old.
…The mean follow-up time since initiating ART was 6.0 (± 3.7) years… (best median p25-p75).
…The mean CD4 counts was 633 cells/µL (± 307); would be better median and p25-p75,
…19 (2%) had HIV-1 RNA levels ≥100,000 copies/mL, and 26 (2%) had LLV. Did the rest of the patients start with a VL < 50 copies/mL?

Reply: Their median age (IQR) was 38 (32-46) years. The median follow-up time since initiating ART was 5.2 (3.1-8.0) years. The mean CD4 counts was 607 cells/µL (434-796).

..14 (1%) had HIV RNA levels ≥100,000 copies/mL, 22 (2%) had LLV, and 875(89%) had VS at switch.

Loss of follow up, deaths and discontinuations must be made according to the change to DTG/3TC or BIC/FTC/TAF. A figure would be recommended
Figure 1: the patients who were switched to DTG/3TC or BIC/FTC/TAF should be divided and in the figure express the number who died, discontinued or were lost to follow-up in each group, as well as the number of DTG VNS /3TC or BIC/FTC/TAF

Reply: Thank for your advice, and we made a change.

Table 1. Table 1, 2 and 4 are correct.
In Table 3, the reference population is unknown. Especially between DTG/3TC and BIC/FTC/TAF.
Reply: We revised Table 3 in the manuscript.


Adverse events and causes leading to discontinuation
It would be advisable to create a Kaplan-Meier curve analyzing the discontinuation rate in each arm (DTG/3TC vs TAF/FTC/BIC).

Reply: We add Fig 3 as Kaplan-Meier curve analyzing the discontinuation rate in each arm (DTG/3TC vs TAF/FTC/BIC).


Discussion

Regarding adverse effects, I would comment that surely many of the patients with ABC/3TC/DTG switched to DTG/3TC, which makes it unlikely that clinicians will attribute side effects to the new regimen.
On the contrary, surely most patients from TAF/FTC/EVG/c were transferred to BIC/TAF/FTC, which makes it more likely to attribute toxicity if it exists. Despite this, no greater toxicity was observed in one arm than the other.
Reply: Thank for your comments, and we found low rate of adverse effects in DTG/3TC and BIC/TAF/FTC and the result was similar.
Insist again on the way in which the analysis is performed at week 48 (Is it a confirmed virological failure, is it a blip or is it a low level replication...?)

Reply: Yes, it is an analysis of snapshot, and our observational cohort was different from randomized clinical trials, which means that we almost didn’t repeat HIV RNA level test within 1 month, because HIV RNA can be measured only 4 times annually according to Taiwan CDC HIV guidelines.

Comment on: HBsAg positive patients should not switch to DTG+3TC due to insufficient HBV control?
Reply: Because only 3 patients with positive HBsAg was switched to DTG/3TC, but 95 patients were switched to BIC/FTC/TAF disproportionally. Moreover, TAF or TDF containing regimens are recommended for HIV positive patients with positive HBsAg status according to Taiwan CDC HIV guidelines.


Comment in the discussion about this…:
…Furthermore, overall adverse effects were generally similar between groups, but increased weight or obesity was the leading cause of discontinuation in patients switching to a regimen of BIC/FTC/TAF than those switching to DTG/3TC… (Comment: some clinicians attribute increased weights to TAF which may justify more adverse effects or discontinuations of BIC/FTC/TAF without solid data based on clinical trials yet.).
Reply: Thank for your valuable comment, and all discontinuations were decided by the discussion between the physicians and patients. The main reason was documented indeed increased weight or obesity in the arm of BIC/FTC/TAF.

Conclusions
In the conclusions I would say something like:
“In conclusion, our study suggests that the effectiveness and safety profiles of switching to DTG/3TC and BIC/FTC/TAF were comparable in treatment-experienced adults with HIV. The presence of high viral loads, frequent virological failure before the switch and positive HBsAg status might impact the benefits of these regimens in the short term of follow-up”
Reply: Thank for your valuable comments, and we excluded positive HBsAg status and HIV RNA > 100,000 copies/mL in logistic regression, because included certain variables in the model that might have collinearity, such as a PVL >100,000 cp/mL, a history of virological failure ≥2 times. Hence, we revised our conclusion as well.

Bibliografía,

I would include a similar study already evaluated in this journal such as:

Mendoza I, Lázaro A, Espinosa A, Sánchez L, Horta AM, Torralba M. Effectiveness, durability and safety of dolutegravir and lamivudine versus bictegravir, emtricitabine and tenofovir alafenamide in a real-world cohort of HIV-infected adults. PLoS One. 2023 Sep 29;18(9):e0291480. doi: 10.1371/journal.pone.0291480. PMID: 37773939; PMCID: PMC10540944.

Reply: Thank for your advice, and we add this article as reference 19.
