# Supplementary material for: Assessment of risk factors for virological nonsuppression following switch to dolutegravir and lamivudine, or bictegravir, emtricitabine, and tenofovir alafenamide fumarate in a real-world cohort of treatment-experienced adults living with HIV
Source: PLoS One. 2024 Nov 20;19(11):e0314003. doi: 10.1371/journal.pone.0314003 (PMC11578452; doi:10.1371/journal.pone.0314003)
Supplement: Attachment — Submitted filename: response to reviewer version 4.docx [file pone.0314003.s003.docx]

Dear Editor,

We would like to thank you and the reviewers for giving us the opportunity to improve our manuscript entitled “**Assessment of risk factors for virological nonsuppression following switch to dolutegravir and lamivudine, or bictegravir, emtricitabine, and tenofovir alafenamide fumarate in a real-world cohort of treatment-experienced adults living with HIV**”. Our point-to-point response to the reviewers' comments and queries are listed below.

**General comments**
Please be consistent with the way the < and > signs are used. For example, in introduction, "HIV RNA levels > 50 copies/mL at 6 months after starting therapy and confirmed HIV RNA levels > 50 copies/mL with previously undetectable HIV RNA levels are defined virological failure [2]." Then in another sentence, "This includes both low-level viremia (LLV, HIV RNA levels <50–1000 copies/mL) and virological failure (VF, HIV RNA levels >1000 copies/mL)." I would suggest deleting the space after the sign as done in the second example, and being consistent throughout the manuscript.

Reply: Thank for your suggestion, and we have revised and made the document consistent according to your recommendation.

**Results**
I would suggest rephrasing to say, "Between October 2019 and January 2023, we first screened 1,086 individuals and then included 988 individuals who switched their ART regimen to BIC/FTC/TAF or DTG/3TC, after excluding those who did not meet the inclusion criteria.

Reply: Thank for your suggestion, and we have revised and made the document consistent according to your recommendation.

Please consider revising the sentence, "It's worth noting that these 7 patients already achieved VS at baseline." It is still unclear how the patients were 7 if there were 3 in each group with K65R. Also please use the words "It is.." rather than "It's ..."

Reply: It is worth noting that these 6 patients already achieved VS at baseline.

For clarity, please include a footnote under table 4 for "completed LTBI treatment, then shift back to previous ART, n (%)", to note that "After completing LTBI treatment, some patients preferred to switch back to their previous ART regimen.

Reply: Thank for your suggestion, and we have revised and made the document consistent according to your recommendation.
